# Self-immunity microcapsules for corrosion protection of steel bar in reinforced concrete

**DOI:** 10.1038/srep18484

**Published:** 2015-12-17

**Authors:** Yanshuai Wang, Guohao Fang, Weijian Ding, Ningxu Han, Feng Xing, Biqin Dong

**Affiliations:** 1 Department of Civil Engineering, Guangdong Provincial Key Laboratory of Durability for Marine Civil Engineering, Shenzhen University, Shenzhen 518060, P.R. China

## Abstract

A novel microcapsule-based self-immunity system for reinforced concrete is proposed. Its feasibility for hindering the corrosion of steel rebar by means of lifting the threshold value of [Cl^−^]/[OH^−^] is discussed. Precisely controlled release behavior enables corrosion protection in the case of depassivation. The release process is characterized over a designated range of pH values, and its release characteristics of the microcapsules, triggered by decreasing pH value, are captured by observing that the core crystals are released when exposed to a signal (stimulus). The aim of corrosion protection of steel bar is achieved through the constantly-stabilized passive film, and its stability is promoted using continuous calcium hydroxide released from the microcapsule, restoring alkaline conditions. The test results exhibited that the release process of the microcapsules is a function of time. Moreover, the release rate of core materials could interact with environmental pH value, in which the release rate is found to increase remarkably with decreasing pH value, but is inhibited by high pH levels.

Reinforced concrete is universally recognized as an outstanding artificial construction material, with excellent mechanical performance and workability. However, its durability is strongly associated with the steel corrosion accompanied by electrochemical reactions. Corrosion in reinforced concrete is often induced by the chloride permeation, and exacerbated by temperature gradients, humidity changes and potential differences^1^. Normally, the embedded steel bar is protected by the surrounding concrete thanks to the passive film formed on its surface to keep it from corroding by providing a highly alkaline environment. However, if the alkalinity locally compromises, for example in the case of chloride permeation, carbonation or sulfation, a high corrosion risk may be initiated, which will be further enhanced by the presence of water (usually entraining harmful ions), oxygen and potential difference. Such processes may significantly shorten the service life of reinforced concrete structures, causing a widespread concern in construction engineering.

Numerous researchers have confirmed that corrosion initiation in reinforced concrete structures is triggered when the [Cl^−^]/[OH^−^] ratio outnumbers certain critical value^2^,^3^,^4^,^5^,^6^,^7^,^8^,^9^,^10^,^11^,^12^,^13^,^14^,^15^,^16^,^17^,^18^,^19^,^20^. Unfortunately, such a threshold cannot get a consensus according to the previous literatures. The [Cl^−^]/[OH^−^] ratio is mentioned earlier by Venu *et al.*^7^, whose results shown that passivation current will be increased when the chloride concentration exceeds hydroxide. Some publications indicate this ratio is a constant value, with around 0.6 by Hausmann^8^ and a stable linear relationship between hydroxide concentration and chloride with a slope of 0.83 by Gouda^9^. Li *et al.*^10^,^11^,^12^ assert that a strong inhibitory effect for corrosion can be obtained due to the significant increase of [Cl^−^]/[OH^−^] ratio by stabilizing hydroxide ions at high levels. These studies are all based on the simulated concrete pore solution, whereas some researchers, such as Glass *et al.*^13^, Andrade *et al.*^14^, Yonezawa *et al.*^15^, Page and Lambert^16^,^17^, Alonso *et al.*^18^, Alonso *et al.*^19^ and Oh *et al.*^20^, who carried out the experiment in cementitious material to investigate the [Cl^−^]/[OH^−^] ratio, argue that it seems to be unreliable to draw conclusions only in solution level. The buffering capacity to alkaline reserve in concrete (such as calcium hydroxide which is slightly soluble in water, and some complex salt, Friedel’s salt^21^) should not be ignored. The bound chlorides are also taken into account if a more accurate [Cl^−^]/[OH^−^] ratio is expressed.

Although there are some divergences in terms of the value of [Cl^−^]/[OH^−^] ratio, the consensus that increasing pH level can delay corrosion initiation can be pointed out. However, the available amount of hydroxyl ions inside concrete is limited due to calcium hydroxide being a hydration product of cement. Conversely, the supply of chloride ions may be effectively unlimited, due to continuous permeation from the surroundings. Additionally, calcium hydroxide may be consumed because of the presence of soluble chlorides, which are often regarded as carriers of chloride ion invasion^22^. Along with the ingress of chloride ions, *CaCl*_*2*_ will react with *Ca(OH)*_*2*_, forming *Ca(OH)*_*2*_*·CaCl*_*2*_*·H*_*2*_*O (*or *CaClOH)*, and the total amount of hydroxide ions will decrease^23^. Calcium hydroxide, with lower solubility, is gradually consumed by calcium hydroxyl chloride (*CaClOH*), which leads to the outflow of hydroxyl ions and decrease of pH value in concrete matrix. Thus, there are two strategies for corrosion protection, decreasing chloride ions or increasing hydroxide ions.

Conventionally, the direct addition of corrosion inhibitors to the reinforced concrete is to bind or combine chloride for reducing the [Cl^−^]/[OH^−^] ratio. However, some drawbacks will appear due to the direct application of corrosion inhibitors. The solubility of a corrosion inhibitor plays a very important role in determining the efficiency of the protection it can provide. Low solubility may result in a poor rust protection effect because of the lack of the active agent, whereas high solubility of corrosion inhibitors will cause pre-mature reactions during hydration of the cement, which could adversely influence the composition and microstructure of the hydration products. Meanwhile, consumption of a certain amount of corrosion inhibitor at an early age leads to less availability of the agent to bind the chloride at later stages.

To address the problems described above, a novel cement-based microcapsule self-immunity system is proposed, which can achieve the high-efficient corrosion protection of steel bar based on the pH self-regulation to decrease the [Cl^−^]/[OH^−^] ratio. The schematic of this system is presented in Fig. 1(a). For comparison, the consequences of ion permeation for a non-functionalized matrix are also illustrated in Fig. 1(b). In this kind of microcapsule, a core agent containing hydroxyl ions is encapsulated in order to prevent pre-mature reactions with other components during the early-age hydration of the cement. More importantly, a pH sensitive shell material is carefully selected to control the release of the hydroxyl ions. The ethyl cellulose (EC)/calcium hydroxide microcapsule satisfies these requirements and is thus suitable for regulating the hydroxide concentration in cementitious materials. The release process of the microcapsules subjected to a series of pH gradients is monitored by means of a micro-plate spectrophotometer.

Also, a chemical trigger mechanism^24^ is highlighted in this paper. Unlike other self-healing methods, which are based on physical trigger mechanisms for the repair of micro-cracks^25^,^26^,^27^, the microcapsule proposed is mainly disintegrated in solutions with low pH value, not at high pH level solution. Their release process is of paramount importance, so the objective of this study is to demonstrate the applicability of such self-immunity microcapsules.

## Results

**Figure 2:**
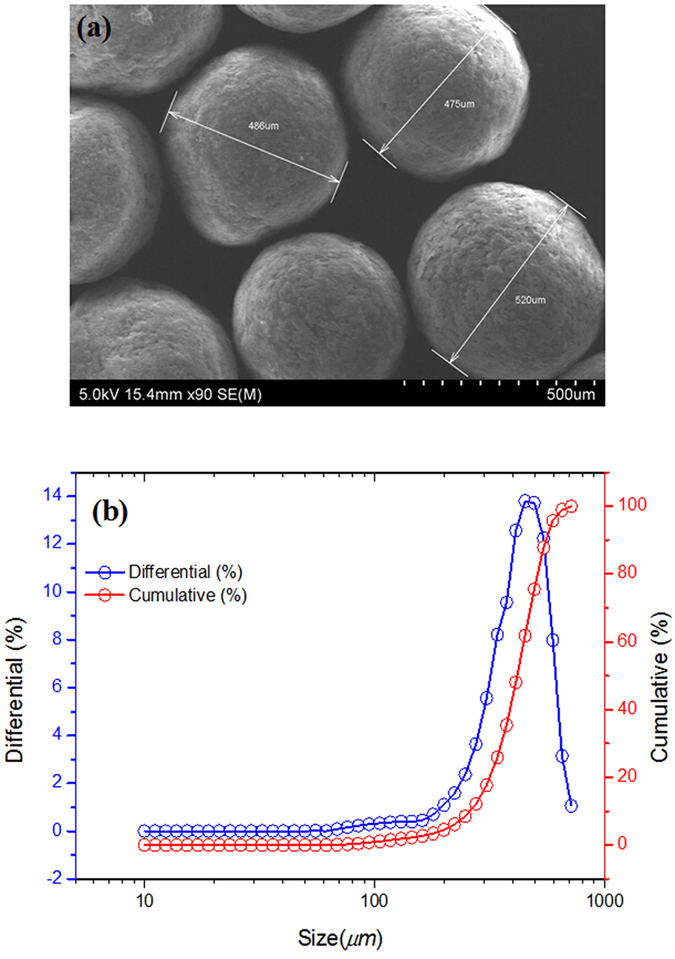
SEM morphology (**a**) and particle size distributions (**b**) for microcapsules. Differential (%) means the percentage of tested particle size, and Cumulative (%) means the cumulative percentage of tested particle size.

Microcapsules can be fabricated using a variety of techniques^28^,^29^, including spray drying, interfacial polymerization, polymer phase separation, layer-by-layer deposition, and membrane emulsification. Although these approaches typically yield microcapsules with polydisperse sizes, they appear basically spheroidal and their diameters are normally distributed^30^; this was the case in our study as shown in Fig. 2(b). Additionally, the morphologies of the intact microcapsules were captured by SEM; typical examples are shown in Fig. 2(a). The experimental results indicate that the capsule diameters generally lie in the range 300 to 700 *μ*m, with a mean diameter of 499.3 *μ*m.

In reinforced concrete structures, deterioration due to corrosion of the embedded steel bars does not occur immediately at the beginning of the service of structures. This implies that corrosion protection may be not necessary during this period. Ideally, forming a persistent passive film on the surface of the steel bars is one of the most desirable properties in terms of corrosion protection in reinforced concrete. If a microcapsule possesses such a property, it is employable in prolonging concrete’s durability, especially in corrosion protection of the embedded steel bar. In order to confirm its feasibility, the experimental results are shown as follow.

**Figure 3:**
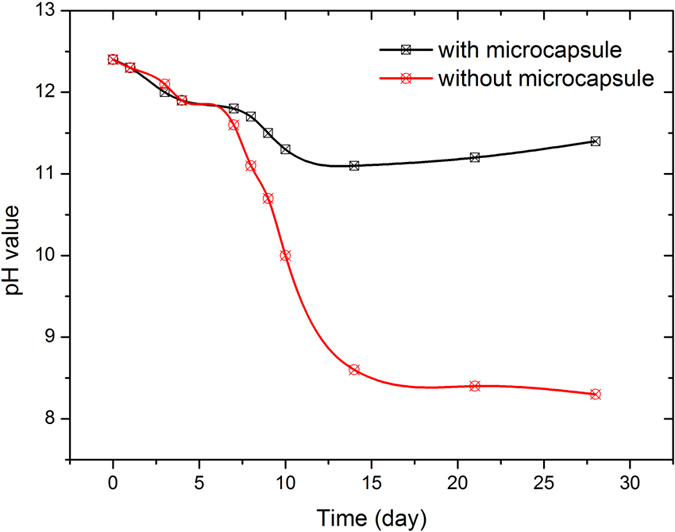
Variation in pH value along with time in saturated calcium hydroxide solutions with or without microcapsules.

In the first place, a comparative trail is demonstrated. The relationship between time (day) and pH value was shown in Fig. 3, describing the variation in saturated calcium hydroxide solutions with and without microcapsules. It seems that both types of solution were found to experience a steady drop in pH value. The pH self-adjustment capacity of the microcapsules becomes apparent around day 6. Afterwards, the curves for the two solutions diverge markedly. The pH for the solution with microcapsule even increased slightly after the 14^th^ day; in contrast, the no-microcapsule solution underwent a monotonic drop in pH throughout the duration of the experiment. This pH recovery feature can be attributed to the pH sensitive capsule shells, contributing to regulate the difference in OH^−^ concentration between the inside and outside of the microcapsules.

In the second place, to further examine the release kinetics of the ethyl cellulose/calcium hydroxide microcapsules, a pH meter and a micro-plate reader were employed to monitor the variation of pH value and the release process of the capsule core, respectively. In our previous paper^31^, a modified EDTA (Ethylene Diamine Tetra-acetic Acid) titration method was adopted to determine the release process of polystyrene resin (PS)/sodium monofluorophosphate (Na_2_PO_3_F, MFP) microcapsules. In this case, such a method could not be implemented due to the different choice of core materials. For the microcapsules discussed in this paper, it is expected that the release process, being a function of time, shows a smart interaction with lowering pH value in the solution. The calcium ion calibration curve of OD produced at 575 nm is utilized to determine the amount of Ca^2+^ released from the capsule cores.

**Figure 4:**
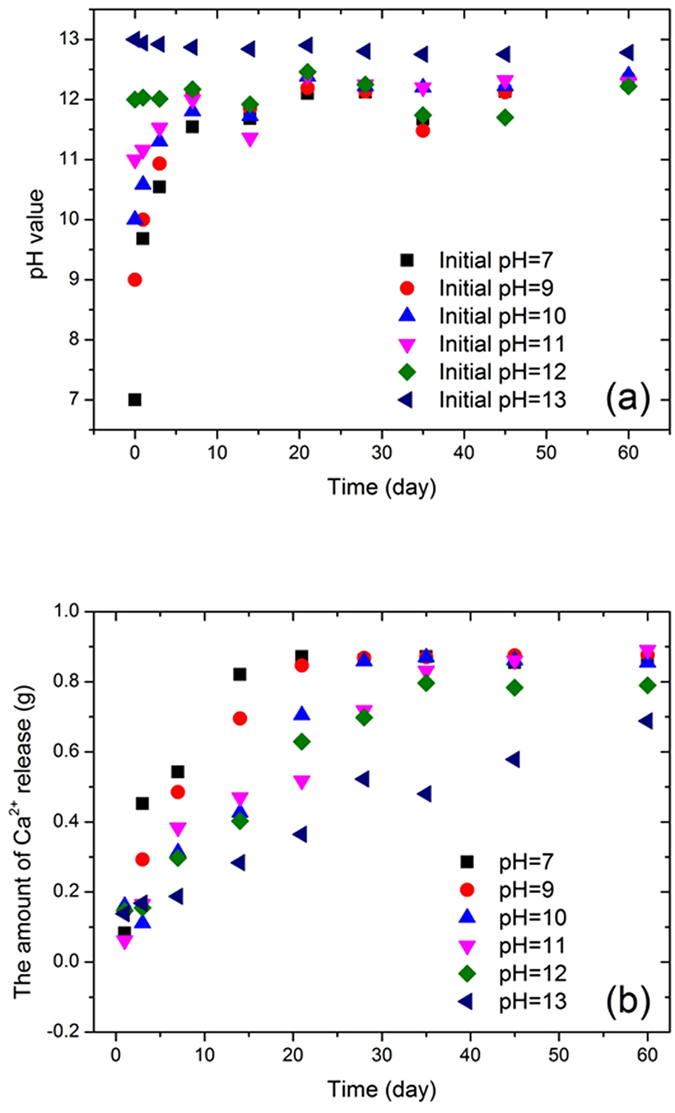
Changes of six initial pH values (**a**) and of calcium ions release amount (**b**) along with time.

According to the calibration curve, the amount of core material released by the microcapsules at every designated pH level was calculated, as the pH value was measured. Figure 4(a) plots the change of pH value over time for different initial pH values. After a 60-day test, the pH value tended to be stable at approximately 12.5 for all adopted initial pH values, although fluctuations appeared during the middle period in some cases. The dramatic change of pH value at the beginning of the tests suggests that core materials were released from inside the microcapsules due to the gradual breaking down of the microcapsules induced by low pH level. This further supports the idea that pH value can be used as a trigger for such a smart release system. The accumulative amount of calcium ion released at different pH values as a function of time is illustrated in Fig. 4(b). The release amount significantly increased with time for all adopted initial pH values, except for the case of pH = 13 which exhibited almost no change in the considered release amount. In addition, the presence of water may cause local swell of EC film, which can be avoided by mixing ethanol and methylbenzene with the ratio of 1:4. Such a formula equips ethyl cellulose with a strong film-forming property due to its low viscosity and high malleability^32^. With regard to the appearance of micro-cracks on the capsule surface, due to a concentration gradient of core material between inside and outside of the microcapsules, the core material in the microcapsules can be released through the micro-cracks even if water is only small amount. At the beginning, the release rate might be slow since there is little damage on the surfaces of microcapsules. As the release process progresses, the number and size of cracks or pinholes increases, resulting in accelerated release. This mechanism can explain the dramatically increased release rates observed in the experiments between 14 days and 28 days. Since higher concentrations of calcium ion correspond to higher pH values, the influence of the microcapsules is obvious. The precipitates are deposited in solution or on the surfaces of the capsules, which can form a dynamic equilibrium between calcium ions and calcium hydroxide. Another effect is that the precipitates may fill the micro-cracks and pinholes, eventually hindering the release of core material. Hence the amount of released material is larger for lower pH values. Under weakly basic or neutral conditions, there are no calcium ions present and the dynamic equilibrium cannot occur. As a result, the release rate is high from the start.

Another point worth mentioning is the reason for choosing calcium hydroxide. The pH value of saturated calcium hydroxide solution tends to be stabilized at over 12.5, which is in favor of corrosion protection of reinforcement. Furthermore, calcium hydroxide powder is only slightly soluble in water, enabling microcapsule to enhance the controlled-release ability. The compatibility between calcium hydroxide and cementitious composites is also need to be emphasized. Unlike other agents, calcium hydroxide is one of hydration products, which neither influences the hydration procedure of cement nor destroys the components and microstructure of cementitious composites.

Thus, the results of release testing indicate that the adopted microcapsules are good candidates for a rust-proofing mechanism in concrete with a smart pH-adjustment feature. The signal of decreasing pH value can be used as a chemical trigger. In addition to chloride permeation, such a trigger is also related to the one of the most commonly occurring degradation phenomena in concrete, namely, carbonation. Carbonation in concrete drags the pH value down to a low level where depassivation of the reinforcement takes place and corrosion imitation is started. Once the trigger signal is activated, the core material in the microcapsules will be released and the alkaline environment of concrete will be gradually rehabilitated.

**Figure 5:**
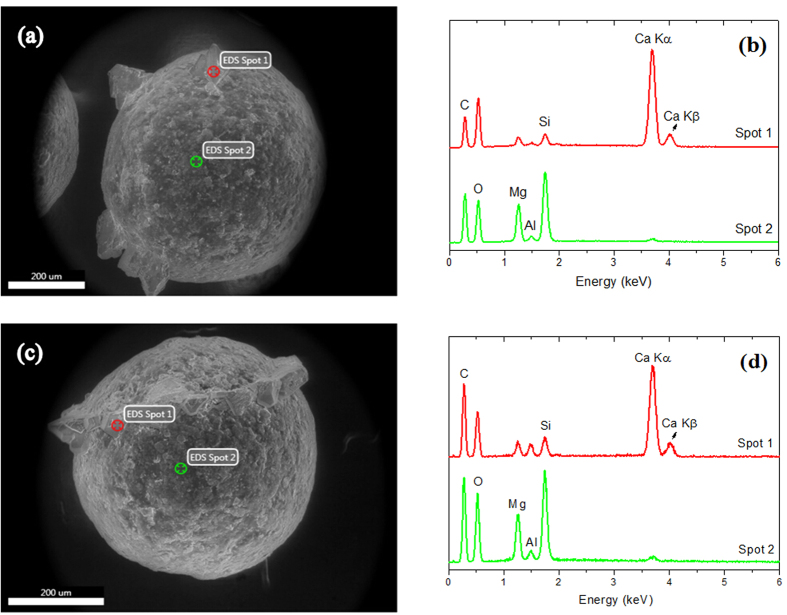
EDS of the triggered microcapsule in solution with pH = 10. (**a**,**b**) are the results at the 7^th^ day; (**c**) and (**d**) are the results at the 28^th^ day.

In the third place, the microscopic and chemical analyses were further used to verify the morphology on the surface of microcapsules triggered at different times and pH values. Since the above-described results only highlight the overall release effect, which means that not all the microcapsules subjected to a given set of conditions (time, pH value) will be chemically triggered, it is imperative to carry out a further investigation. ESEM equipped with EDS (energy dispersive X-ray spectroscopy) was performed to observe release characteristics of the microcapsules^33^; Fig. 5 exhibits the characteristics of typical microcapsules in solution with pH = 10 at days 7 and 28. Comparing the ESEMs, a far denser structure of crystal production can be seen at the 28^th^ day than the 7^th^ day. Scattered pinholes are relatively prominent in Fig. 5(a), whereas the sample in Fig. 5(c) is dominated by long, narrow crevices. In the case of designated solutions whose alkalinity is prepared using different amounts of sodium hydroxide, crystal production appears on the surface of the microcapsules. This might be calcium hydroxide, since it has low solubility in water. However, there are no calcium ions in the solution, which denotes that such crystal productions only come from the microcapsules. In contrast, the region without crystals might not be chemically triggered. The above inferences are further verified by means of EDS. The EDS results shown in Fig. 5(b,d) demonstrate that calcium hydroxide is the main component in the crystal production zone and the region without crystals includes the elements of shell material. Moreover, various peaks indicate a high level of elemental content. The significant difference between the two spots is in the relative amounts of calcium, silicon and magnesium. Silicon and magnesium is the major elemental composition of the shell material, which includes talcum powder (the main component of which is magnesium silicate), while calcium comes from the calcium hydroxide encapsulated in the microcapsules. Such test results are consistent with highly favorable release characteristics.

**Figure 6:**
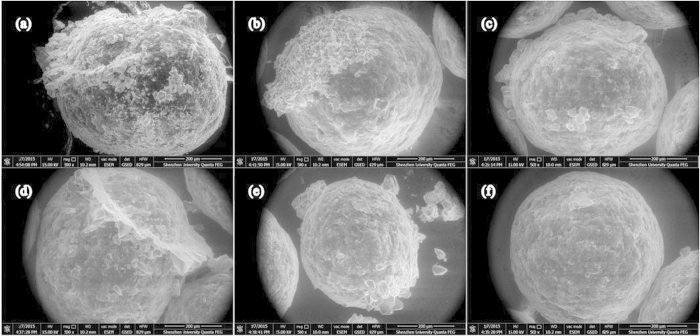
ESEM images of microcapsules after soaking in solutions with different pH for same soaking time. pH = 7, 9, 10, 11, 12, 13 are displayed in (**a**–**f**), respectively.

Finally, the release characteristics at different times are morphologically compared. Figure 6 shows ESEM images of effective microcapsules after soaking in solutions with different pH values for equal periods of time. In the case of pH = 13, there is no obvious crystal growth on the surface of the soaked microcapsules. As the pH value is reduced, surface damage becomes more and more obvious. Moreover, morphological features including points (pinholes), lines (crevices) and planes (peeling) develop as the pH value is lowered, and are especially noticeable for the neutral environment with pH = 7 where the microcapsule surface is almost totally penetrated by points, lines and planes after soaking. These traits further confirm that environmental pH has a significant effect on the microcapsule release behavior, and can be effective as a trigger mechanism for this type of self-immunity system.

## Discussion

To summarize, the microcapsule-based self-immunity system has been found to be a promising prospect for corrosion protection applications, which is ensured by smart interaction between the system’s [Cl^−^]/[OH^−^] value and corrosion initiation. Our strategy of re-alkalization efficiently functionalizes the reinforced concrete, realizing pH self-adjustment.

For microcapsule fabrication, one should note the materials of the core and shell, which are the main functional representation. Another point is the basic properties of microcapsules, namely, size and appearance. These factors also contribute to the functionalization of the microcapsules.

The release process and release characteristics of the microcapsules are strongly emphasized in this paper. The release investigation indicates that such a self-immunity microcapsule can be triggered by low pH values, and calcium hydroxide can be controllably released to regulate the environmental pH condition. Additionally, ESEM observation analysis was conducted at different times and pH values. Test results show that the microcapsule release process is a function of time. Moreover, the release rate of core materials could interact with environmental pH value; the rate increases markedly with decreasing pH value, but is inhibited by high pH values.

## Methods

### Microcapsule preparation

Micron-sized capsules were fabricated via the extrusion-spheronisation and spray drying method. First of all, hydroxyl propyl methyl cellulose (HPMC), polysorbate 80 and microcrystalline cellulose (MCC) were mixed into calcium hydroxide in the ratio 2:2:39:57 as the core materials, which were fabricated to be core particle by means of extrusion-spheronisation. HPMC and polysorbate 80 were used as the adhesive agents and MCC acted as the grafted skeleton. Secondly, ethyl cellulose (EC) is dissolved into the solvent (ethanol and methylbenzene with the ratio of 1:4). Such a solution is shell material. Solvent of shell material will be evaporated to the air in this process, and the EC will cover on the surface of core particles. The slight addition of talcum powder is to avoid adhesion among the microcapsules, but the release kinetics of microcapsules is not linked to talcum powder.

### Experimental scheme on controlled-release features

1) Two equivalent saturated calcium hydroxide solutions were used to simulate a concrete environment. 5 g calcium hydroxide powder was mixed into one solution, whereas about 10 g microcapsules (the amount of calcium hydroxide in microcapsules nearly equalizes with 5 g calcium hydroxide powder), in contrast, was added into the other one. The pH value of solution was sampled at designated times. 2) Since the microcapsules are designed for use in reinforced concrete structures for the rust protection of steel bars based on pH self-regulation, understanding their release process under different conditions is of paramount importance. For this purpose, solutions with different pH values (pH = 7, 9, 10, 11, 12 and 13) were prepared using matched quantities of sodium hydroxide. Ethyl cellulose/calcium hydroxide microcapsules were added to the solutions with a 5% mass fraction. Polyethylene film was used to cover the mouth of each beaker to prevent the penetration of carbon dioxide. The amount of released calcium ions was quantified with a micro-plate spectrophotometer. O-cresolphthaleincomplexone (OCPC) and adenosine monophosphate (AMP) were selected as calcium ion indicators, and the optical density (OD) produced at 575 nm in the color reaction was obtained by a micro-plate reader. The amount of calcium ions released from the capsule core was calculated according to the calcium ion calibration curve of OD at 575 nm. The spectrophotometer method was carried out three times for each specimen to avoid systematic errors.

### Experimental apparatus

The micro-morphology and particle size distribution were captured by a scanning electron microscope (SEM) (SU-70, Hitachi, Japan) and a laser particle analyzer (LPA) (BT-9300ST, Bettersize, China), respectively. An environmental scanning electron microscopy (ESEM) (Quanta FEG 250, USA) equipped with texture element analysis microscopy (TEAM) (EDAX, USA) was used to observe the release characteristics of the microcapsules.

## Additional Information

**How to cite this article**: Wang, Y. *et al.* Self-immunity microcapsules for corrosion protection of steel bar in reinforced concrete. *Sci. Rep.* **5**, 18484; doi: 10.1038/srep18484 (2015).

## Figures and Tables

**Figure 1:**
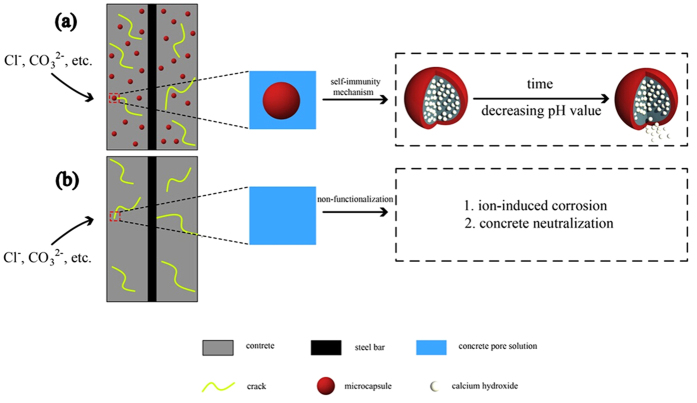
(**a**) Schematic of microcapsule-based self-immunity system; (**b**) possible consequences of unprotected matrix in the case of ion penetration.
